# Correlation between magnetic resonance images of peritumor margin enhancement and prognosis in hepatocellular carcinoma after drug-eluting bead transcatheter arterial chemoembolization

**DOI:** 10.3389/fonc.2023.957710

**Published:** 2023-04-04

**Authors:** Donglin Kuang, Nan Zhang, Mengfan Zhang, Hao Li, Xinwei Han, Jianzhuang Ren, Xuhua Duan

**Affiliations:** Department of Interventional Radiology, The First Affiliated Hospital, Zhengzhou University, Zhengzhou, Henan, China

**Keywords:** hepatocellular carcinoma, drug-eluting bead transcatheter arterial chemoembolization, magnetic resonance imaging, margin enhancement, morphology

## Abstract

**Purpose:**

The aim of this study is to investigate the morphological characteristics and clinical significance of magnetic resonance (MR) images of peritumor margin enhancement in hepatocellular carcinoma (HCC) after drug-eluting bead transcatheter arterial chemoembolization (DEB-TACE).

**Methods:**

From January 2017 to December 2020, a total of 162 patients who received a diagnosis of HCC were included in our study. We began the follow-up with magnetic resonance imaging (MRI) for complete response assessment, and peritumor margin enhancements were classified as sharp and rough types according to morphology. During the follow-up, data such as progression or remission of the two enhancement modalities, morphological changes in terms of margin enhancements observed in MR images, and alpha-fetoprotein (AFP) levels were recorded.

**Results:**

In the follow-up period of 36 months, 70 and 92 patients with sharp- and rough-type peritumor margins, respectively, were observed. At the end of the follow-up, patients with sharp-type margins had lower AFP levels and longer progression-free survival than those with rough-type margins (*P* < 0.05). Furthermore, the sharp-type margin was thinner than the rough-type margin (all *P* < 0.05). Moreover, the sharp-type group had a high incidence of tumors with a diameter of < 5 cm, whereas the rough-type group had a high incidence of tumors with a diameter of ≥ 5 cm. Continuous enhancements of peritumor margins in MRI were greater in the sharp-type group than in the rough-type group. Most of the patients with a sharp-type margin achieved disease remission (94.3%, *P* < 0.05), whereas most of those with a rough-type margin experienced disease progression (84.8%, *P* < 0.05).

**Conclusions:**

Patients with HCC with a sharp-type margin enhancement on MRI after DEB-TACE mostly demonstrated benign lesions with a good prognosis, whereas those with a rough-type margin mostly demonstrated malignant growth.

## Introduction

Transcatheter arterial chemoembolization (TACE) is an important treatment methodology for hepatocellular carcinoma (HCC). In recent years, drug-eluting bead TACE (DEB-TACE) has demonstrated a clear advantage in HCC treatment (1, 2). However, neither TACE nor single-cycle therapy with DEB-TACE can effectively control primary HCC. Repeat therapy with on-demand TACE based on postoperative tumor response was considered effective in prolonging the overall survival of patients with primary HCC (3, 4). Therefore, determining when to provide on-demand TACE treatment in patients with primary HCC is crucial.

However, the Response Evaluation Criteria In Solid Tumors (RECIST) criteria cannot accurately determine efficacy based on changes in tumor size (5). The modified RECIST (mRECIST) criteria focused on evaluation based on tumor enhancement, making the tumor response assessment accurate and intuitive. Enhanced computed tomography (CT) and magnetic resonance imaging (MRI) after TACE and DEB-TACE in patients with HCC showed different morphological enhancements of peritumor margins (6, 7). Although tumor diagnosis can be made directly through puncture biopsy pathological detection for this type of margin enhancement, the uncertainty of invasive and small lesions makes it unsuitable for the assessment of margin enhancement after HCC surgery. Although positron emission tomography/CT can be used to assess tumor activity, it has limitations, such as high application cost and poor recognition ability with differentiated tumors, making its application in the local evaluation of HCC after TACE unfeasible (8). Owing to the high sensitivity and high popularity of MRI, we chose MRI in this study for the morphological analysis of peritumor margin enhancement after TACE.

Whether the enhancement of different morphological margins around the tumor on MRI means that the tumor has recurred after DEB-TACE is unknown. The evaluation of these enhancements was relevant to determine the therapeutic effect and whether further clinical intervention was needed. In our previous study, we find that the sharp-type peritumor margins occur more frequently in those primary HCC patients with tumor size < 5cm after DEB-TACE treatment, whereas the rough-type peritumor margins occur more frequently in those primary HCC patients with tumor size ≥ 5cm (9); however, it is not clear whether the post–DEB-TACE peritumor margins by MRI would affect the prognosis of these HCC patients. Hence, this study with a long-term follow-up was further carried out, which was based on the morphology of peritumor margin enhancement observed through MRI after DEB-TACE, and the morphological differences and progression of different types of peritumor margin enhancements were compared and analyzed to provide the theoretical basis for the evaluation of tumor response after DEB-TACE and the requirement of repeated TACE therapy.

## Methods

### Study design and participants

From January 2017 to December 2020, the data of patients with HCC were reviewed and analyzed by the Department of Interventional Radiology of the First Affiliated Hospital of Zhengzhou University. The clinical data of the patients are presented in **Table 1**. The inclusion criteria were as follows: (1) met the clinical diagnostic criteria for primary HCC or had histopathological results confirming HCC; (2) had complete data of tumor enhancement (MR images) and alpha-fetoprotein (AFP) ≥ 200 at 1 week before DEB-TACE, 1–6 months of follow-up, and the end of follow-up; (3) had a preoperative Eastern Cooperative Oncology Group score of ≤ 2 points, with Child–Pugh grade A or B; (4) had a single tumor in the liver; and (5) had unimpeded blood circulation of the portal vein trunk and the left/right branches. The exclusion criteria were as follows: (1) MR images with respiratory motion artifact interference; (2) cancerous thrombus and/or thrombosis causing occlusion of the portal vein trunk or the left and right branches of the portal vein; (3) previous local treatments other than DEB-TACE; and (4) multiple tumors in the liver.

**Table 1:** Baseline characteristics of the study population.

Variable	HCC patients (N=162)
Age (years)	
Range	28-81
Mean ± SD	58.0 ± 10.0
Age for males (years)	
Range	28-81
Mean ± SD	58.0 ± 9.3
Age for females (years)	
Range	31-79
Mean ± SD	57.9 ± 12.4
Sex, N (%)	
Male	127 (78.4)
Female	35 (21.6)
Hepatitis C infection, N (%)	127 (78.4)
Hepatitis B infection, N (%)	28 (17.3)
Other disease origins, N (%)	7 (4.3)
ECOG grade, N (%)	
0	86 (53.1)
1	61 (37.7)
2	15 (9.2)
Child-Pugh class, N (%)	
A	112 (69.1)
B	50 (30.9)
Maximum diameter of the largest tumor (cm), N (%)	
<5	47 (29.0)
≥5	115 (71.0)
>10	34 (21.0)

HCC, hepatocellular carcinoma; SD, standard deviation; ECOG, Eastern Cooperative Oncology Group.

### DEB-TACE operating procedure

The patients were asked to lay supine on the digital subtraction angiography examination bed. Then, they were administered a preoperative infusion of dezocine and palonosetron. DEB-TACE was performed conventionally. A modified version of the Seldinger technique was used, which involved the puncture of the right femoral artery and placement of a 5F vascular sheath. Transheaths were introduced into the aqueous membrane guidewire and 5F liver tube, and the catheter and duct wire were combined to place the tip of the catheter at the opening of the abdominal trunk artery or were superselected into the hepatic artery. The parameters of the high-pressure syringe were adjusted as follows: flow rate of 5–8 ml/s and total contrast medium (iodixanol 320 mgI/ml) of 10–15 ml. After the arterial angiography of the tumor supply, microcatheters and microwires were inserted interchangeably. After superselected artery to the target blood vessel, CalliSpheres microspheres with a diameter of 100–300 µm (Suzhou Hengrui Callisyn Biomedical Technology Co., Ltd, Suzhou, Jiangsu province, China) loaded with 40–60 mg of adriamycin or epirubicin were used for chemoembolization until tumor staining disappeared. If the tumor was found to have collateral arteries supplying blood (diaphragm artery, superior mesenteric artery, renal artery, etc.), the same method was adopted for embolization after angiographic confirmation. If tumor staining remained after CalliSpheres microspheres were used, additional Embosphere microspheres with a diameter of 300–500 µm (Merit Medical, South Jordan, Utah, USA) were added until the blood flow in the artery supplying blood to the tumor almost stagnated.

### MRI acquisition

The superconducting 3.0 T MR750 MR scanner (U.S. GE) and eight-channel body-part-dedicated phased array coil were adopted. With the xiphoid process as the center, the scan covered the region from the upper margin of the liver to the lower margin. The patients were trained before the examination to cooperate with breathing and to keep the breathing amplitude and frequency consistent by breathing calmly. The first routine sequence scan was performed, and the multistage dynamic enhanced scan used three-dimensional liver acquisition with volume acceleration. The parameters used were as follows: repeat time: 3.7 ms; echo time: 1.6 ms; inversion time: 5.0 ms; visual field: 42 mm × 42 mm; layer thickness: 4.4 mm; and flip angle: 12°. The contrast medium used was gadolinium-diethylene triamine pentaacetic acid of 0.1 mmol/kg with an injection flow rate of 3 ml/s. Respiratory gating techniques were used to scan the arterial and portal veins and delayed phases at 30, 60, and 180 s after injection.

### Image analysis

The computer medical image storage and transmission system performed multipane, multiperiod scanning image linkage reading and repeatedly compared the characteristics of tumor enhancement in different MR image sequences to observe the morphology of tumor margin enhancement. The change in size and shape of the tumor was analyzed through the comparison of preoperative and postoperative imaging data. The imaging manifestations, intensification periods, and morphological changes of each tumor were recorded in each stage of preoperative and postoperative MRI flat scan and dynamic enhancement. The maximum preoperative tumor diameter was measured on the three-phase enhanced MRI image with multidimensional sections, the upper and lower levels of the enhancement level with the sharpest and widest range were selected, and the thickness of the reinforced margin was measured linearly in two dimensions. We calculated the average of the three levels measured as the final result. Double-blind reading was performed by two physicians experienced in MRI evaluation. If the results were inconsistent, a third physician read the images again, and the final result was obtained after discussion.

### Follow-up and efficacy evaluation

**Figure 1:**
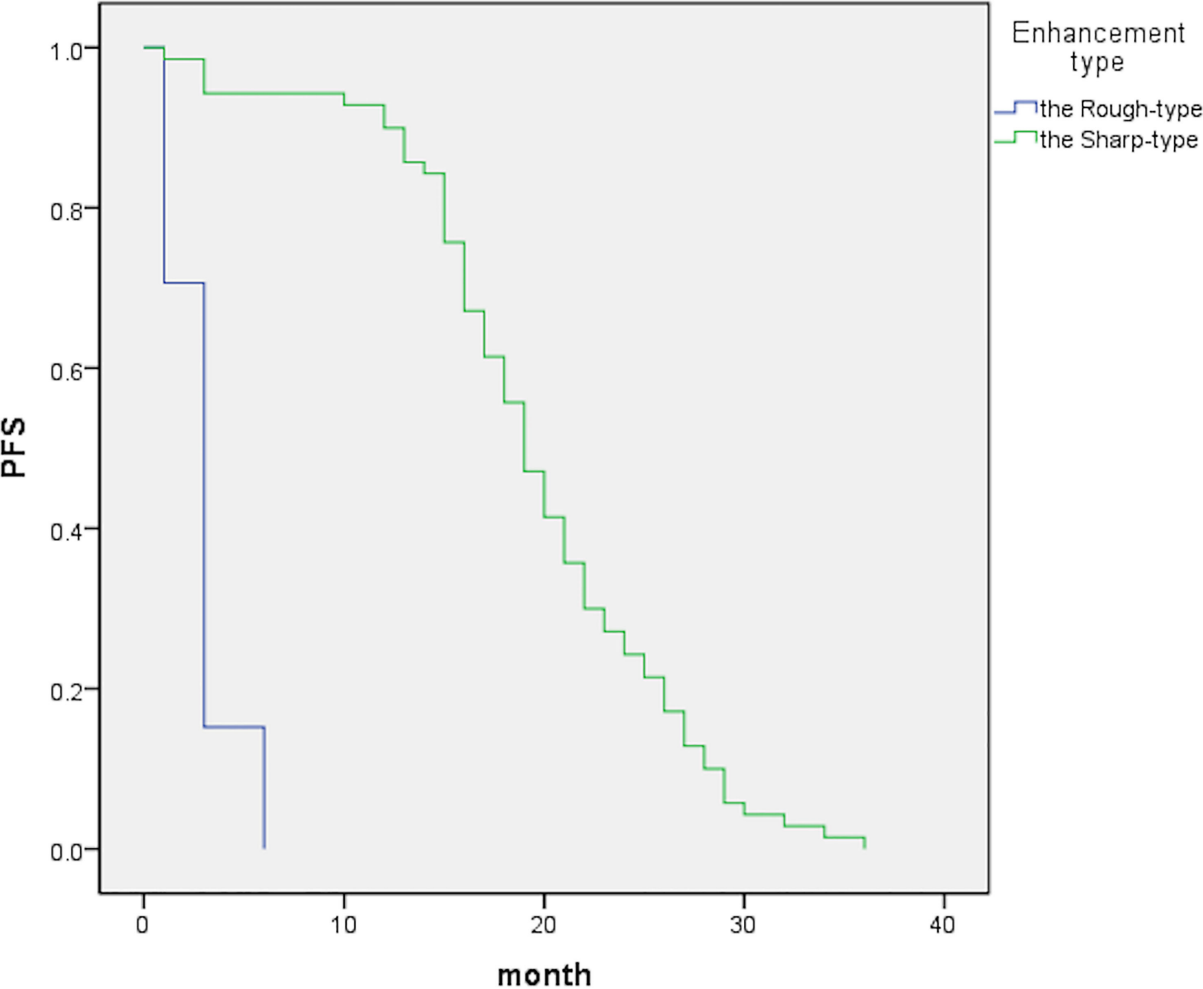
The graph indicates median PFS periods of 19 [95% confidence interval (CI), 17.4–20.6] and 3 (95% CI, 2.7–3.3) months for the sharp- and rough-type groups, respectively.

**Figure 2:**
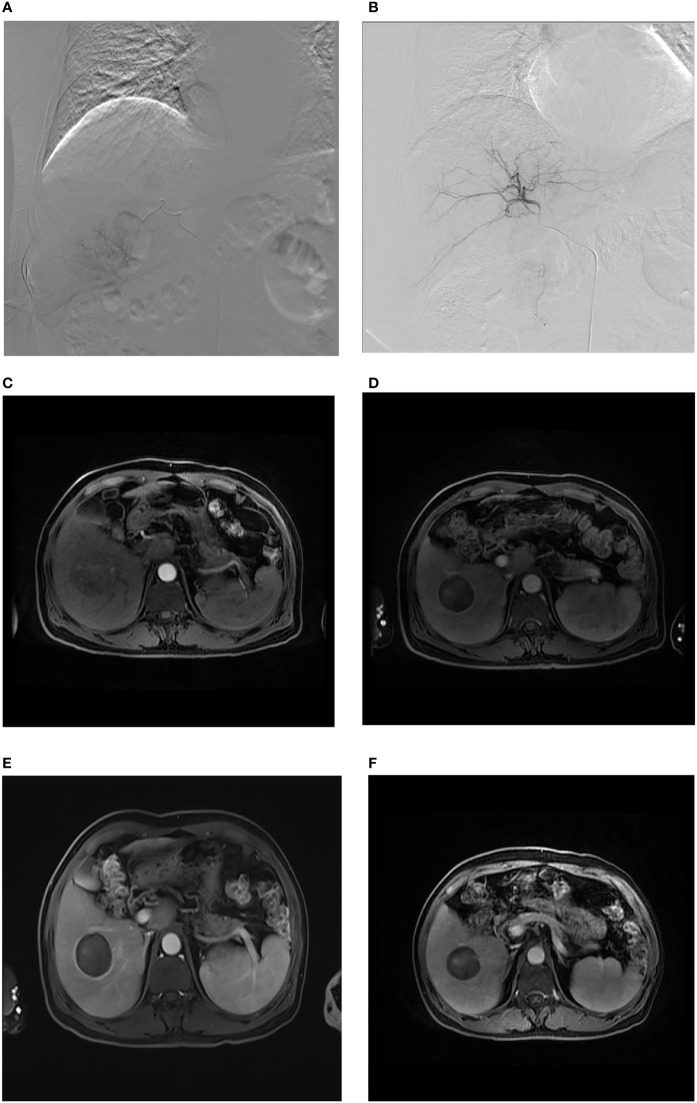
Sharp-type tumor margins observed on an axial MR image. A male patient, aged 61 years, was admitted to our hospital due to the detection of liver lesions in the right lobe of the liver during physical examination. **(A)** When DEB-TACE was administered for the first time, superselective catheterization was performed in the tumor feeding artery, and arteriography demonstrated abnormal focal hyperchromatism at the arterial phase, presenting the “holding ball sign” (white arrow); **(B)** abnormal staining disappeared, as observed through contrast reexamination after the first administration of DEB-TACE; **(C)** as expected, nonhomogeneous enhancement of HCC was indicated at the arterial phase (white arrow). **(D)** One month after the first administration of DEB-TACE, sharp-type peritumoral rim enhancement (black arrow) appeared at the delayed phase and at the beginning of the follow-up. **(E)** Three months after the start of the follow-up period, clear sharp-type peritumoral rim enhancement (black arrow) appeared at the delayed phase. **(F)** Twelve months after the start of the follow-up, the sharp-type peritumoral rim enhancement (black arrow) still existed, but the enhancement was lighter than that in **(E)**.

**Figure 3:**
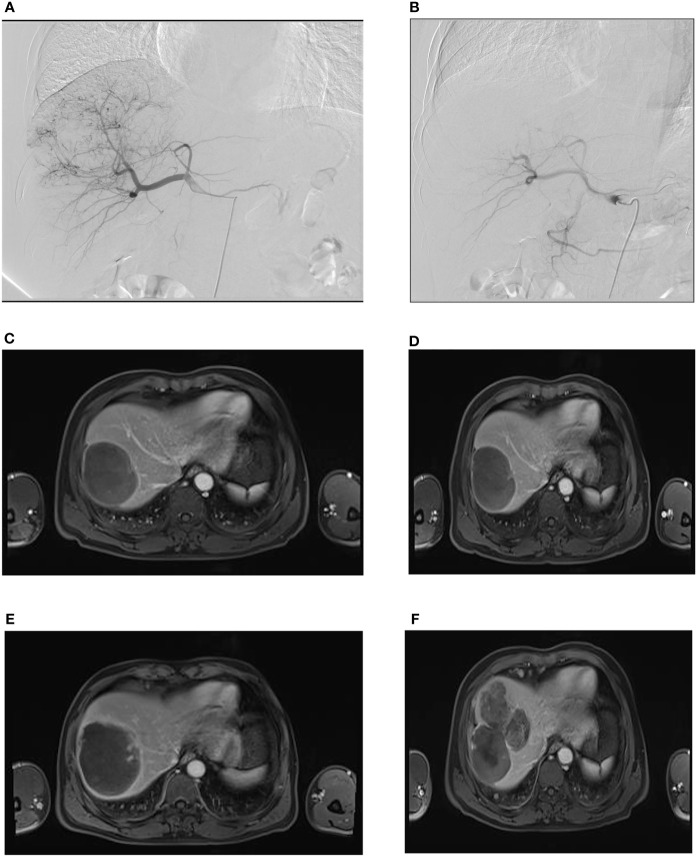
Rough-type tumor margins observed on an axial MR image. A male patient, aged 55 years, was admitted to our hospital with a complaint of right upper abdominal pain for 3 days. **(A)** During the first administration of DEB-TACE, hepatic arteriography showed massive abnormal hyperchromatism in the right posterior lobe of the liver. **(B)** Abnormal hyperchromatism disappeared at the end of the arterial phase, as observed through contrast reexamination after DEB-TACE; **(C)** the patient was included in the follow-up after the second administration of DEB-TACE, and MR images showed a rough-type margin on the left side of the tumor (black arrow). **(D)** One month after the start of the follow-up period, the rough-type margin (black arrow) was slightly larger than that in **(C)**. **(E)** Three months after the start of the follow-up, the thickness of the rough-type margin (black arrow) increased and invaded the surrounding area (white arrow). **(F)** Six months after the start of the follow-up period, the tumor completely recurred in the corresponding direction (white arrow, black arrow) of the rough-type margin in **(E)**.

MRI was reviewed 1 month after the first DEB-TACE, 1.5–2 months after the second DEB-TACE, and 2 months after the third DEB-TACE. Patients were assessed for complete response (CR) through MRI evaluation once every 2–3 months. Follow-up was initiated when the MRI evaluation revealed CR after 1–3 DEB-TACE sessions. During the follow-up period, peritumor margin enhancements were classified as sharp and rough types according to morphology. Patients with persistent CR during the follow-up were considered to have disease remission based on the mRECIST criteria. Patients who showed a change from CR to partial response (PR), stable disease (SD), or progressive disease (PD) according to the mRECIST criteria were considered to have disease progression, and the follow-up was terminated. In this study, progression-free survival (PFS) was defined as the duration of sustained CR status of lesions according to the mRECIST criteria. We collected data on the thickness of peritumor margin enhancement, delayed enhancement, and disease progression or remission and recorded differences in PFS (**Figure 1**). Based on numerously previous studies (6, 10–17), peritumor margin enhancement morphology in MRI was defined as follows: (1) sharp-type peritumor margin (**Figure 2**): smooth enhancement along the margin throughout or at an arc of the necrotic tumor, which is uniform, thin, and sharp, with a clear demarcation between the tumor and liver; (2) rough-type peritumor margin (**Figure 3**): rough or blurred (streak) enhancement, which is irregular or serrated, with endogenous and convex enhanced nodules. Furthermore, if the rough-type peritumor margin and sharp-type peritumor margin coexisted in one patient, he/she was classified as having a rough-type peritumor margin.

In terms of imaging, disease recurrence was defined as imaging results after tumor treatment showing new enhancements compared with those pretherapy. Some scholars advocated the use of “local progression” to describe recurrent disease (11). On the basis of all observations in this study, we summarized three morphological changes in the margins for progressive enhancement: (1) increase in ranges (including length and thickness), (2) increase in the enhancement degree, and (3) irregularity in morphology. Margin enhancements for remission were as follows: (1) narrowed ranges (including length and thickness), (2) no obvious morphological change, (3) disappearance of enhancement, (4) weakened enhancement degree, and (5) more regular morphology. Compared with the baseline morphological pattern of the margin enhancement shown in MR images obtained at the initiation of the follow-up, the presence of (1) or (2) and (3) in the corresponding MR images was considered to be local tumor progression, and any of (1), (2), and (3) or both (4) and (5) suggested local tumor remission.

### Statistical analysis

SPSS 20.0 statistical software was used for data analyses and figure plotting. The measurement data are expressed as the mean ± standard deviation ( $\bar{x}$ *± s*). The Kaplan–Meier method and log-rank test were used to compare PFS between the two groups, and the differences in AFP levels and margin thickness were compared using two independent samples *t*-tests. The classification data were tested using the χ^2^ test. *P* < 0.05 was considered statistically significant.

## Results

In total, 162 patients were included in the analysis and followed up for 3–36 months. Among them, 70 and 92 patients had sharp- and rough-type peritumor margins, respectively. The mean PFS of the patients with the sharp-type margin was 19.5 ± 7.0 months, whereas that of the patients with the rough-type margin was 2.9 ± 1.6 months (*P* < 0.05, **Table 2**). The enhancement of the sharp-type margin was less than that of the rough-type margin (*P* < 0.05, **Table 3**). After DEB-TACE, the sharp-type group had a higher incidence of tumors with a diameter of < 5 cm, and the rough-type group had a higher incidence of tumors with a diameter of ≥ 5 cm. No significant difference was observed between tumors with diameters of 5–10 and > 10 cm (*P* > 0.05, **Table 4**). The rate of sustained enhancement was higher in the sharp-type group than in the rough-type group (88.6% vs. 33.7%; *P* < 0.05, **Table 4**). The remission rate was higher in the sharp-type group than in the rough-type group (94.3% vs. 15.2%; *P* < 0.05, **Table 4**). At the beginning of the follow-up, no significant difference was observed between the sharp- and rough-type groups in terms of AFP levels (26.6 ± 16.5 ng/ml vs. 29.4 ± 17.8 ng/ml; *P* > 0.05). At the end of follow-up, the sharp-type group had lower AFP levels than the rough-type group (21.0 ± 14.7 ng/ml vs. 513.6 ± 255.9 ng/ml; *P* < 0.05).

**Table 2:** Comparison of PFS between two types of peritumoral rim enhancement.

Peritumoral rim enhancement	Sharp-type (*n*=70)	Rough-type (*n*=92)	*P (t)*
PFS (month), mean ± SD	19.5 ± 7.0	2.9 ± 1.6	0.00 (146.916)

**Table 3:** Comparison of thickness between two types of the peri-tumor edge enhancement by contrast-enhanced MRI in HCC patients after DEB-TACE (mm, $\bar{x}$ *± s*).

Peritumoral rim enhancement	Start of follow-up	3 months of follow-up	*P (t)*
Sharp-type (*n*=70)	3.5 ± 1.6	3.4 ± 1.7	0.54 (0.614)
Rough-type (*n*=92)	11.7 ± 4.5	19.2 ± 6.1	0.00 (6.530)
*P(t)*	0.00(12.040)	0.00(14.170)	

**Table 4:** Comparison of indexes between two types of the peri-tumor edge enhancement by contrast-enhanced MRI in HCC patients after DEB-TACE (%).

Peri-tumor edge enhancement	Tumor diameter				3 months of follow-up		3 months of follow-up	
	<5 cm	≥5cm			Continuous enhancement	Non-continuous enhancement	Progression	Remission
		5~10 cm	>10cm	Total				
Sharp-type (*n*=70)	39(55.7)	26	5	31(44.3)	62(88.6)	8(11.4)	4(5.7)	66(94.3)
Rough-type (*n*=92)	8(8.7)	63	21	84(91.3)	31(33.7)	61(66.3)	78(84.8)	14(15.2)
*P* value	<0.05				<0.05		<0.05	

## Discussion

In this study, patients with HCC had different peritumor margin enhancement morphologies, and the progression rate also varied. The nonenhancement areas in enhanced imaging were highly correlated with pathologically confirmed tumor necrosis and were considered imaging markers of successful treatment (5, 10). Analyses of the imaging data of the postoperative tumor area of local treatment have revealed that the thin and smooth margin enhancements around the tumor showed better outcomes, usually with not only a thickness of 1–2 mm but also 5 mm, which can last for 6 months and generally disappears approximately 3 months after a single operation (6, 11, 18). However, in this study, tumors with the sharp-type margin did not disappear after 3 months of follow-up, which may be related to multiple DEB-TACE treatments (11). In addition, the sharp-type margin was thinner than the rough-type margin at the beginning of follow-up and after 3 months. Studies have suggested that AFP levels are associated with postoperative recurrence of HCC (19). At the beginning of the follow-up, both sharp- and rough-type margins achieved CR. No active tumor tissue components were found, AFP levels were low, and no significant difference was observed between the two types. At the end of the follow-up, AFP levels were lower in the sharp-type group but higher in the rough-type group. Furthermore, the PFS of the sharp-type group was significantly longer than that of the rough-type group after achieving CR. Patients with sharp-type margins who have reached remission are still being followed up to date. The rough-type group had malignant growth potential, whereas the sharp-type group was likely to have a good prognosis.

Studies have shown that tumor size is closely related to the CR rate after TACE. The smaller the tumor, the easier it is to achieve CR (20). Studies have shown that tumors < 5 cm had a higher rate of CR after DEB-TACE than tumors ≥ 5 cm (21). In this study, tumors < 5 cm were prone to have sharp-type margins, whereas those ≥ 5 cm were prone to have rough-type margins after DEB-TACE. A difference was observed in tumor sizes between the two types of peritumor margin enhancements. We believe that this is related to the tumor size, load, and aggressiveness. After DEB-TACE, large tumors were prone to residual tumor and recurrence, and small tumors were likely to be completely necrotic. However, no significant difference was observed in the incidence of the two peritumor margin enhancements between tumors of 5–10 cm and those of > 10 cm. This may be related to the relatively insufficient sample size of tumors > 10 cm.

Pathological examination of the margins of smooth peritumor tissues did not reveal cancer cells but revealed inflammatory cell infiltration and granulation tissue hyperplasia. Compared with pathological tissues, thin and smooth peritumor margin enhancement causes complete necrosis of most lesions (11, 22, 23). HCC is susceptible to microvascular invasion (MVI). MVI is an independent risk factor for recurrence and metastasis of HCC. The MVI of the peritumor tissue in HCC has been the pathological basis for the formation of nonsmooth tumor margins (17, 24). Studies have shown that nonsmooth tumor margins predict MVI with up to 90% sensitivity and specificity. The larger the tumor, the more likely it is to develop MVI (24, 25). This was consistent with the higher frequency of the occurrence of the rough-type margin with a tumor diameter of ≥ 5 cm in this study. In addition, in this study, the continuous enhancement rate of the MRI delay period of the sharp-type margins was significantly higher than that of the rough-type margins. We believe that this was related to the delayed clearance of the contrast medium by benign tissue and hyperplasia in the “border” area of tumor tissue and normal liver tissue after the complete necrosis of tumor tissue (26). Abundant tumor microvessels were observed on the margin of the rough-type peritumor, and they had the imaging characteristics of HCC. A small amount of sustained enhancement may be because HCC with MVI had small tumor emboli blocked in the tiny branches of the surrounding portal vein. Compensatory hyperperfusion of local hepatic arteries occurred in areas of decreased and absent portal vein blood flow (24).

The MR images of the peritumor margins after HCC treatment with DEB-TACE can be classified as sharp- and rough-type margins according to morphology. Our long-term follow-up studies have shown that tumors with a sharp-type peritumor margin have benign lesion characteristics and indicate good prognosis after DEB-TACE in patients with HCC. Conversely, tumors with a rough-type peritumor margin showed more growth and aggressive potential and indicated malignant growth. In addition, scholars have achieved similar results in the enhancement morphology of metastatic hepatic malignancy (10, 11, 27). Perhaps these two types of morphologies used in this study are universal for hepatic malignancies, but this needs to be confirmed through further studies.

Some novelty in this study could be noticed. Recently, some novel technologies have been proposed to numerically analyze morphology in HCC patients. Lambin et al. first proposed radiomics as a new technology in 2012, whose main purpose is to quantitatively extract the image features through high-throughput algorithms and then identify and predict tumor heterogeneity (28). However, only a few studies explore the application of radiomics in evaluation of tumor morphology in HCC patients receiving TACE, which is mainly limited by the following reasons: (a) a non-neglectable proportion of HCC patients present as invasive lesions, which makes it difficult to identify the boundary of tumors by region of interest (ROI) drawing by machine learning, while the heterogeneity among radiologists using artificial labeling is also inevitable; hence, how to accurately ROI is the first question in applying radiomics in HCC (29); (b) bleeding and necrosis in liver cancer lesions also negatively affect the extraction of radiomics features in the liver (30). (c) HCC in different countries or regions presents huge heterogeneity (31), which makes it difficult to establish a unified radiomics model. In contrast, the method applied in this study is less affected by the above mentioned conditions, and it also presents a high repeatability, which is easy for the clinician to learn and can help them to rapidly make the treatment decisions; furthermore, it might help to shorten the outpatient-waiting time for HCC patients.

This study had the following limitations. First, this study was retrospective. Therefore, newer imaging methods, such as hepatobiliary MRI with ethoxy-benzyl-diethylenetriamine pentaacetic acid, could not be evaluated. Second, different practitioners control the embolization extent in DEB-TACE differently. This may have affected the morphological characteristics of HCC on MR images of enhancements. In addition, this study was a single-center study; hence, it is necessary to conduct a multicenter study and establish a multicenter MRI evaluation expert panel to maintain the authenticity and reliability of the results. Furthermore, perfusion images in MRI about the peritumor margin in HCC patients receiving DEB-TACE are still lacking, which is our future study direction.

This study showed that the morphology of enhancement of the sharp-type and rough-type peritumor margins can estimate the prognosis of DEB-TACE treatment in HCC. This enabled the diagnosis of the peritumor as benign or malignant based on MR images after DEB-TACE in HCC, which helped clinicians determine the treatment opportunity of TACE on demand.

## Data availability statement

The raw data supporting the conclusions of this article will be made available by the authors, without undue reservation.

## Ethics statement

The studies involving human participants were reviewed and approved by The First Affiliated Hospital, Zhengzhou University. Written informed consent for participation was not required for this study in accordance with the national legislation and the institutional requirements.

## Author contributions 

XD and JR designed the work; DK wrote the main manuscript text; DK, NZ, HL, and MZ prepared the acquisition, analysis, and interpretation of data; XD and DK prepared figures and tables. All authors reviewed the manuscript. All authors contributed to the article and approved the submitted version.
